# The development of the Internal Resource Perception Scale: Validity and reliability

**DOI:** 10.1371/journal.pone.0348075

**Published:** 2026-04-29

**Authors:** Ratana Saipanish, Suwannee Putthisri, Soontaree Srikosai, Phuangphet Kaesornsamut, Pattaraporn Pandee, Thanavadee Prachason, Thiprawee Chattrattai

**Affiliations:** 1 Department of Psychiatry, Faculty of Medicine Ramathibodi Hospital, Mahidol University, Bangkok, Thailand; 2 Rajanagarindra Institute of Child Development, Department of Mental Health, Ministry of Public Health, Chiang Mai, Thailand; 3 Department of Mental Health and Psychiatric Nursing, Faculty of Nursing, Mahidol University, Nakhon Pathom, Thailand; 4 Southern Institute of Child and Adolescent Mental Health, Phunphin, Surat Thani, Thailand; 5 Department of Masticatory Science, Faculty of Dentistry, Mahidol University, Bangkok, Thailand; Universidad Santiago de Cali, COLOMBIA

## Abstract

**Background:**

The perception of internal resources influences mental well-being and unlocks the potential for personal growth. However, there is currently no tool that directly addresses perceptions of internal resources. This cross-sectional psychometric study aimed to develop a tool for assessing personal perceptions of internal resources in a Thai context.

**Methods:**

The Internal Resource Perception Scale (IRPS) was developed through a comprehensive literature review, a focus group interview, and the research team’s expertise. Content validity was assessed by experts in inner growth and psychotherapy. A convenience sample of 514 Thai adults participated in an empirical examination of the scale. Exploratory factor analysis (EFA) was used to determine the factor structure, and construct validity was examined using Spearman’s correlations between IRPS scores and other validated psychological measures.

**Results:**

The scale content validity index (S-CVI) of the 42-item IRPS was  .94, with item content validity index (I-CVI) values ranging from  .80 to 1.00. The EFA and item reduction identified 25 items loaded onto four factors: Compassionate and Ethical Nature, Adaptable Mindset, Responsible Spirit, and Logical Perspective, explaining 62.4% of the observed variance. Confirmatory factor analysis (CFA) supported the four-factor structure, while a bifactor model indicated a strong general factor underlying the scale. The scale showed excellent internal consistency (McDonald’s omega = .96). The IRPS scores demonstrated positive correlations with measures of well-being (Resilience Inventory, ρ = .58; Revised Rosenberg Self-Esteem Scale, ρ = .37; World Health Organization-Five Well-Being Index, ρ = .38) and negative correlations with measures of distress (Patient Health Questionnaire, ρ = −.41; Generalized Anxiety Disorder-7, ρ = −.34).

**Conclusions:**

The IRPS demonstrates strong psychometric properties and can serve as a useful tool for understanding how individuals perceive their own internal resources. It can help individuals improve their self-awareness, learning, and personal growth. Its applicability in clinical settings should be explored in future research.

## Introduction

Internal resources are frequently mentioned and widely recognized in psychology, yet the concept remains unclearly defined. Generally, internal resources refer to psychological characteristics that help individuals cope with challenges, promote resilience, and improve their well-being [1,2]. These may include curiosity, self-efficacy, emotional regulation, self-esteem, optimism, courage, patience, compassion, inner strength, firmness, connectedness, flexibility, and spirituality [1–7]. Several theoretical frameworks emphasize the importance of internal resources in psychological adaptation. For example, the Satir model proposes that each individual inherently possesses the internal resources necessary for coping and growth [8,9]. Resilience theory highlights internal resources as key protective factors that support individuals in bouncing back from adversity [10]. Positive psychology focuses on cultivating inner strengths and self-awareness as pathways to flourishing and optimal functioning [11]. Post-traumatic growth theory suggests that individuals often emerge stronger from adversity when they can access and recognize their inner psychological capacities [12]. Similarly, Wade-Bohleber et al. described internal resources as intrapsychic characteristics that contribute to coping and psychological stability [13].

While existing research has explored internal resources through related concepts such as resilience, personal strength, and self-concept [6,14–17], it is important to distinguish between having internal resources and being aware of them. Many previous studies have tried to assess the presence or strength of psychological traits but have paid less attention to whether individuals are aware of or perceive the resources they possess within themselves. People differ not only in the psychological characteristics they possess but also in how well they are aware of, perceive, acknowledge, and utilize these resources [18–20]. Some individuals readily recognize their internal resources, while others overlook, undervalue, or fail to acknowledge them. Some people need a supportive process to achieve self-awareness and acceptance [21].

While existing measurement tools−such as the Inner Strength Questionnaire [15], Brief Resilience Scale [22], and Self-Transcendence Scale [23]−assess internal resources as traits or behaviors, they do not explicitly attempt to measure an individual’s self-perceived internal resources. However, recognizing one’s internal resources is a crucial psychological process that influences self-efficacy, resilience, and overall well-being. Individuals who perceive themselves as having more internal resources tend to demonstrate greater adaptability, mental health, and coping abilities [24–28], whereas those who see themselves as lacking inner resources can experience self-doubt, distress, and diminished psychological resilience [29–31].

Self-perception, particularly regarding internal resources, also plays an important role in the healing process [11,32,33]. As individuals begin to recognize and value their own resources, they become more inclined and capable of addressing and working on their problems or weaknesses [34]. However, there is currently no tool that directly addresses how individuals perceive their internal resources. This study aimed to develop and validate a psychometric tool specifically designed to assess individuals’ self-perceptions of internal psychological resources. By focusing on perceived internal resources, this scale offers a novel contribution to psychological assessment.

## Materials and methods

### Study design and participants

This study employed a cross-sectional survey. Because it targeted the general population rather than a specific subgroup, participants were recruited using convenience sampling from four regions of Thailand (Central, Northern, Northeastern, and Southern) to efficiently capture diverse backgrounds. The participants were recruited through posters and personal contacts. Demographic information (gender, age, educational level, occupation, and marital status) was collected to provide a comprehensive description of the sample. The recruitment period for this study was 1 November 2023–15 January 2024.

The inclusion criteria were individuals over 18 years of age who could communicate, read, and understand the Thai language fluently. This language ability was assessed during the recruitment process by inviting potential participants to read the study’s purpose and informed consent documents, followed by brief questions to confirm their understanding. Participants who did not demonstrate sufficient language comprehension or could not complete the questionnaires were excluded from the study. Those who met the inclusion criteria and provided informed consent were asked to complete the survey.

We aimed to recruit 500 participants, which would exceed the recommended minimum sample size for factor analysis of 10–20 times the number of questionnaire items [35–37]. Based on the initial number of items, a minimum of 450 participants was recommended. Thus, our planned sample size would be considered more than adequate, even prior to item reduction. After being informed about the research objectives, processes, and confidentiality principles, all participants provided informed written consent and then completed a questionnaire that gathered general information, the Internal Resource Perception Scale (IRPS), and other study measures. This study was conducted in accordance with the established ethical principles for human research in the Declaration of Helsinki. It was reviewed and approved by the Ethical Committee of the Faculty of Medicine, Ramathibodi Hospital, Mahidol University, Bangkok (Approval No. COA.MURA2023/508).

#### Instruments.

The Resilience Inventory-9 (RI-9) is a measure designed by Wongpakaran et al. [14] to capture the extent to which an individual can recover after encountering trouble or a setback. Its creation was based on the basic concept of inner strength, conceptualized as an internal psychological capacity that enables individuals to endure adversity, regulate emotional responses, and recover from stress, and which was theoretically derived from the Buddhist concept of the Ten Perfections. It comprises nine questions for self-assessment with the respondent selecting one of five possible responses (from 1 = “does not describe at all” to 5 = “it describes me very well”). The scale has demonstrated excellent internal consistency (Cronbach’s alpha = .91) and validity, showing positive correlations with the inner strength-based inventory and negative correlations with anxiety, somatization, and depression scores [14]. In the current sample, the RI-9 similarly exhibited excellent internal consistency (Cronbach’s alpha = .95, McDonald’s omega = .95).

The revised version of the Rosenberg Self-Esteem Scale (RSES-TR) is a tool used to assess self-esteem, developed by Rosenberg et al. [38] and translated into Thai by Wongpakaran et al. [39]. It consists of five positive and five negative questions (a total of 10 items). Each question has four possible response options (“strongly agree,” “agree,” “disagree,” and “strongly disagree”). In previous research, the tool has demonstrated good internal consistency (Cronbach’s alpha = .86). In the current sample, the RSES-TR exhibited good reliability (Cronbach’s alpha = .89, McDonald’s omega = .89).

The World Health Organization-Five Well-Being Index (WHO-5) is a questionnaire that measures current mental well-being, developed by the World Health Organization and translated into Thai by Saipanish et al. [40]. It consists of five questions, with six possible responses to each (from 0 = “never” to 5 = “all the time”). This measure has demonstrated good internal consistency (Cronbach’s alpha = .87) and the ability to detect depression based on scores <12 with a sensitivity of  .89 and a specificity of  .71 [40]. In the current sample, the WHO-5 exhibited excellent internal consistency (Cronbach’s alpha = .94, McDonald’s omega = .95).

The Patient Health Questionnaire-9 (PHQ-9) is a self-rated scale, developed by Kroenke et al. [41] and translated into Thai by Lotrakul et al. [42], that assesses depressive symptoms. It comprises nine questions, each with four answer options (from 0 = “never” to 3 = “almost every day”). The instrument has demonstrated acceptable internal consistency (Cronbach’s alpha = .79) and was able to identify depressive symptoms with a score ≥ 9, exhibiting a sensitivity of  .84 and a specificity of  .77 [42]. In the current sample, the PHQ-9 exhibited excellent internal consistency (Cronbach’s alpha = .90, McDonald’s omega = .90).

The Generalized Anxiety Disorder-7 (GAD-7), developed by Spitzer et al. [43], is a self-rated scale that assesses anxiety symptoms. It consists of seven questions, each with four response options (from 0 = “never” to 3 = “almost every day”). The tool has exhibited excellent internal consistency (Cronbach’s alpha = .92) and was able to detect anxiety with a score ≥ 10, demonstrating a sensitivity of  .89 and a specificity of  .82 [43]. In the current sample, the GAD-7 exhibited good internal consistency (Cronbach’s alpha = .89, McDonald’s omega = .89).

### Development of the Internal Resource Perception Scale

#### Item development.

The research team extensively reviewed various journals, documents, books, and online sources to gain insight into the internal resources of individuals. Searches in Google Scholar and PubMed used the keywords “inner resources,” “internal resources,” “psychological resources,” human*, child*, and adult* (with “*” allowing matches of additional wildcard characters for additional forms of those search terms). Subsequently, a focus group interview was conducted with members of the general population to gather a wide array of perspectives on “internal resources.” This focus group comprised eight individuals–three men and five women–aged between 25 and 53 years and recruited through convenience sampling based on availability. The 90-minute focus group session took place in a private room without interruptions. After outlining the meeting’s objectives, the facilitator asked open-ended questions, such as “When you hear the term ‘internal resources’ of a person, what comes to your mind?” and “Why do you think these qualities are important?” to encourage in-depth sharing.

From the literature review, focus group, and research team’s expertise in psychotherapy, a list of potential resource-related items was compiled. At this stage, no predefined theoretical factor structure was imposed, and the items were generated inductively to ensure comprehensive coverage of internal resources. All items were thoroughly examined and analyzed, and redundant or overlapping items were eliminated according to consensus among the research team members. Following this process, the IRPS was drafted, resulting in an initial pool of 45 internal resource items (S1 Table). This tool had a main question for respondents to assess the extent to which they perceived their internal resources by completing the statement “I am…” Each item was formulated as an adjective representing a resource, such as “compassionate,” “receptive,” “flexible,” and so forth, aiming to evoke a sense of self-awareness (i.e., the state of the respondent). A 5-point Likert scale, with responses ranging from 0 (not at all) to 4 (a lot), was chosen to enhance interpretability and user-friendliness. Before proceeding to content validation, the initial 45 items were reviewed internally by the research team. This internal review focused on the clarity of wording, appropriateness of response format, and coverage of the intended content domains.

### Data analysis

#### Assessment of validity.

**Content validity:** The tool was revised and checked for content validity by five experts who were carefully selected based on their specialization in inner growth. In this research, inner growth refers to psychological development processes that enhance self-awareness, emotional regulation, resilience, and personal meaning–core components of internal resources [44,45]. Because inner growth is closely linked to internal resources, we have confidence that experts with extensive working experience in mental healing and psychotherapy can effectively evaluate the relevance and accuracy of the internal resource items. The experts were four psychiatrists and a psychiatric nurse who all had at least 20 years of clinical experience as psychotherapists; four of them had been working in an academic setting, and three had a research background.

They independently assessed the level of relevance for each item’s corresponding construct on a four-point scale from 1 (irrelevant) to 4 (relevant), and provided qualitative comments regarding the clarity, redundancy, and conceptual fit of each item. Kendall’s coefficient of concordance was used to calculate the degree of agreement among the raters. The item content validity index (I-CVI) and scale-level content validity index (S-CVI) were then calculated.

Regarding face validity, the revised IRPS was reviewed by 20 representatives from the general population, who were invited through convenience sampling. Participants were asked to independently read and complete the scale and then provide feedback, both in written form and verbally. The research team reviewed all suggestions collectively and made wording adjustments when necessary. For example, one item, initially phrased as “มีอิสระ” (“having freedom”), was revised to “มีอิสระภายใน,” which can be translated into English as “free-spirited.”

### Item reduction and internal factor structure

As the instrument was being developed, it underwent item reduction to make the scale as simple as possible while maintaining its positive measurement properties. This was done by using EFA to identify and retain only the most relevant items based on their factor loadings and conceptual clarity. EFA was deemed appropriate because the IRPS was in its initial stage of development, and the underlying factor structure had not yet been fully established.

Before conducting EFA, we evaluated whether the data were suitable for factor analysis. Bartlett’s test of sphericity was used to test the hypothesis that the correlation matrix is an identity matrix [46]; a significant result (p < .05) indicates that sufficient correlations exist among items to justify factor extraction. The Kaiser–Meyer–Olkin (KMO) measure assesses sampling adequacy by quantifying the proportion of shared variance among items [47]; values above 0.60 are commonly considered acceptable for factor analysis, with ≥0.70 indicating good adequacy. These tests therefore served as prerequisites to ensure that the data met the fundamental assumptions for EFA.

Factor extraction was then performed based on principal axis factoring. A promax rotation was employed, as the factors were assumed to be correlated. We used κ = 4 because it is the conventional default in SPSS and is widely recommended in the literature as a reasonable choice for achieving a simple interpretable structure in promax rotation [48,49]. The number of factors to be extracted was determined by parallel analysis (PA) (50) and Velicer’s minimum average partial (MAP) test [50,51]. The eigenvalues extracted from real data that exceeded the 95th percentile of those extracted from random data and the lowest average square partial correlation indicated the number of factors to retain based on PA and MAP test, respectively.

Model selection was considered based on model fit, factor structure simplicity, and theoretical convergence. A number of goodness-of-fit metrics– the Chi-square (χ^2^) test, root mean square error of approximation (RMSEA) [52], Comparative Fit Index (CFI) [53], Tucker–Lewis index (TLI) [54], Standardized Root Mean Square Residual (SRMR) [55], and Bayesian information criterion (BIC) [56]–calculated from Stata’s structural equation modeling framework were considered to determine the best-fitting model among the different factor solutions. In favoring a simple structure, we retained items with primary factor loadings ≥ .40, as this threshold is commonly considered to provide an acceptable level of practical significance for interpreting factors. Items with substantial cross-loadings (i.e., secondary loadings > .30 or a difference between the primary and alternative loadings < .20) were removed because such items are likely to measure more than one construct and may reduce the interpretability of the factors. These thresholds are widely recommended in psychometric practice [57]. Once no more items needed to be removed, confirmatory factor analysis (CFA) was conducted to examine the factor model and model fit of the final scale, using the Weighted Least Squares Mean and Variance Adjusted (WLSMV) estimator. The criteria for acceptable model fit were RMSEA < .08, CFI > .90, TLI > .90, and SRMR < .08 [58]. To ensure that the extracted factors in the final model are distinct, average variance extracted (AVE) was computed from standardized CFA loadings, and the Fornell-Larcker criterion [59] was applied to assess discriminant validity among the factors. If the square root of the AVE of each factor was higher than the factor correlation between them, the two factors were considered distinct [59].

To explore an alternative internal structure, a bifactor model was also tested to evaluate the extent to which item responses reflected a general internal resources factor alongside specific domain factors. The bifactor model specified one general factor on which all items loaded and orthogonal specific factors corresponding to the identified domains.

### Relations to external measures

Five established psychological instruments theoretically related to the construct of internal resources were used to evaluate the construct validity of the IRPS. For convergent validity, the IRPS was expected to correlate positively with resilience (RI-9), self-esteem (RSES-TR), and well-being (WHO-5). For discriminant validity, we used measures of depression (PHQ-9) and anxiety (GAD-7), and hypothesized that the IRPS would be negatively correlated with their scores. Because the IRPS scores and external measures deviated from normality, Spearman’s correlations were used to examine convergent and discriminant validity. Confidence intervals for Spearman’s rho were obtained using nonparametric bootstrapping with 5,000 resamples.

### Assessment of reliability

The internal consistency of the final IRPS and its subscales derived from EFA was determined by McDonald’s omega [60], as it is considered a more accurate reliability estimate than Cronbach’s alpha when item loadings vary or when the sample data violate normality [61–65]. However, Cronbach’s alpha, item-total correlations and item-rest correlations were also recalculated to support the internal consistency reliability as estimated by McDonald’s omega. For the bifactor model, omega hierarchical (ωₕ) was computed to quantify the proportion of reliable variance attributable to the general factor, and omega specific (ωₛ) was calculated to estimate reliable variance unique to each specific factor beyond the general factor. These indices were used to inform interpretation of the total score and subscale scores.

### Statistical software

Analyses of content validity and internal factor structure, including exploratory factor analyses (EFA), PA, and MAP test, were conducted using the Statistical Package for Social Sciences software (version 18.0; IBM Corp.). Stata SE 16.1 was used to perform goodness-of-fit tests, assess discriminant validity of the extracted factors, conduct CFA, and evaluate relations to external measures and reliability.

## Results

A total of 514 individuals participated in the study, slightly exceeding the planned target of 500 due to additional individuals expressing interest during recruitment. Approximately 70% of the participants were female. The average age was 38.24 years (SD = 15.19). Most participants (74.32%) were employed, 55.06% had completed a bachelor’s degree, and 51.95% were single. The sample was evenly distributed across Thailand’s four main regions: North, Northeast, Central, and South. Descriptive statistics of the PHQ-9, GAD-7, WHO-5, RI-9, and RSES-TR are shown in Table 1.

**Table 1:** Characteristics of the participants (n = 514).

Variables	n (%)
Female	360 (70.04)
Occupation	
Unemployed	27 (5.25)
Student	83 (16.15)
Employed	382 (74.32)
Retired	22 (4.28)
Education	
Elementary level	41 (7.97)
High school	113 (21.98)
Graduate degree	283 (55.06)
Post-graduate degree	77 (14.98)
Region	
Northern	127 (24.71)
Northeastern	128 (24.90)
Central	138 (26.85)
Southern	121 (23.54)
Marital status	
Single	267 (51.95)
Couple	194 (37.74)
Divorce /separated	44 (8.56)
PHQ-9, median (IQR)	4 (1-8)
GAD-7, median (IQR)	4 (2-7)
RI-9, median (IQR)	37 (34-42)
RSES-TR, median (IQR)	31 (28-34)
WHO-5, median (IQR)	16 (12-20)

n = number; IQR = Interquartile Range; RI-9 = Resilience Inventory; RSES-TR = Rosenberg Self-Esteem Scale, Thai Revised; WHO-5 = WHO-5 Well-Being Index; PHQ-9 = Patient Health Questionnaire; GAD-7 = Generalized Anxiety Disorder Scale.

### Content validity

The panel of expert judges comprised four psychiatrists and one psychiatric nurse, all with a minimum of 20 years of clinical experience in psychotherapy. Four experts were affiliated with academic institutions, and three had research experience. The experts independently rated all 45 initial items. The Kendall’s coefficient of concordance indicated a moderate level of agreement among the raters (W = .487, χ² (4) = 87.692, *p* < .001).

The item scores obtained from the experts’ evaluations were computed to determine the I-CVI and the S-CVI. The I-CVI values for the IRPS ranged from  .60 to 1.00. The I-CVI was calculated by dividing the number of experts who rated each item as either 3 or 4 by the total number of experts. Based on Lynn’s criteria [66], a minimum agreement threshold of >  .78 was adopted. No item had borderline values (between  .70 and  .79). Three items (“disclose,” “frugal,” and “romantic”) with low I-CVI values (≤ 0.60) were removed. After removing these three items, the I-CVI values ranged from  .80 to 1.00 (S2 Table). The entire scale of 42 items was then revised based on the comments and suggestions of experts. The S-CVI was calculated by taking the sum of the I-CVIs divided by the number of items (i.e., average method). The overall S-CVI for the 42-item scale was  .94, indicating excellent content validity.

### Item reduction and internal factor structure

Exploratory factor analysis was performed to identify the underlying factors of the remaining 42 items. The descriptive statistics of these items are shown in S2 Table. The results show that the data were suitable for EFA because the Kaiser–-Meyer–-Olkin (KMO) coefficient was  .965, and the Bartlett’s sphericity test was statistically significant (χ^2^ = 17143.46, df = 861, *p* < .001).

To determine the number of factors, the PA and MAP tests of the 42-item IRPS (S3 Table) suggested that four and five factors should be retained, respectively.

The EFA revealed that the five-factor solution had a less complex structure, with fewer cross-loadings (alternative factor loadings > .3 or a difference between the primary and alternative loadings > .2) and fewer inadequate primary factor loadings (<  .4) than the four-factor model (S2 Table). Even though the third, fourth, and fifth factors explained < 5% of the variance (S2 Table), the model fit statistics indicated that the five-factor solution [χ² (809) = 3755.91, *p* < .001; χ²/df = 4.64;  RMSEA [90%CI] =  .084 [.082,  .087]; CFI = .825; TLI = .814, BIC = 37116] fit the data better than the other solutions (S4 Table). Therefore, we performed item reduction based on the five-factor model and deleted two items with a primary factor loading < .4 (“sensitive” and “self-aware”) and thirteen items with an alternative factor loading > .3 or a difference between the primary and alternative loadings > .2 (“gratitude,” “independent,” “gentle,” “sincere,” “curious,” “lively,” “compassionate,” “strong,” “emotionally stable,” “calm,” “self-assured,” “humoring,” and “holistic”).

Reapplying the MAP test on the remaining 27 items suggested a four-factor solution (S5 Table), aligning with the fact that the fifth factor in the initial EFA had only one item (“challenge loving”) remaining and should be dropped (S3 Table). However, the PA (S5 Table) suggested retaining two factors. A subsequent EFA of the 27-item scale revealed that the two-factor model exhibited many cross-loadings, suggesting under-factoring (S6 Table). The model fit indices suggested that the four-factor solution fit the data better [χ² (318) = 1250.15, *p* < .001; χ²/df = 3.93; RMSEA [90%CI] =  .076 [.071,  .080]; CFI = .907; TLI = .898; SRMR = .051; BIC = 23524] than the two-factor solutions (S7 Table); therefore, the four-factor solution was selected.

Extracting four factors showed that “challenge loving” and “honest” had low factor loadings and high cross-loadings on the fourth factor, respectively, which were then removed (S6 Table).

The third round of PA and MAP test on the remaining 25 items suggested two- and four-factor solutions, respectively (S8 Table). While the two-factor model yielded many items with high cross-loadings (S9 Table) and explained only 55.6% of the total variance, the four-factor model showed adequate primary factor loadings (range:  .504−.942) without significant cross-loadings (Table 2), explaining 62.4% of the total variance. However, the fourth factor contained only three items and accounted for 2.7% of the variance, raising concerns about its stability, reliability, and practical relevance. Therefore, we performed EFA to extract three factors as an alternative and found that “intelligent” and “organized” (which previously belonged to Factor 4) were combined into Factor 3, but “analytical” fell into Factor 1 instead, with an inadequate primary factor loading (<  .4) and high cross-loading onto Factor 3 (S9 Table). The goodness-of-fit tests also revealed that the four-factor model had acceptable fit indices [χ² (269) = 970.88, *p* < .001; χ²/df = 3.61; RMSEA [90%CI] =  .071 [.067,  .076]; CFI = .924; TLI = .916; SRMR = .044; BIC = 21516] and fit the data better than the two- and three-factor solutions (S10 Table), and was therefore selected.

**Table 2:** Descriptive statistics and factor loadings of the final IRPS (25 items) derived from EFA.

Resources “I am…”	Factor loadings			
	Compassionate and Ethical Nature	Adaptable Mindset	Responsible Spirit	Logical Perspective
Loving	**.942**	.060	−.133	−.049
Caring	**.894**	−.004	.067	−.071
Empathetic	**.841**	−.032	.127	−.054
Easy-going	**.820**	.123	−.207	.024
Conscientious	**.747**	−.065	.211	.003
Humble	**.645**	−.056	.221	.019
Faithful	**.538**	−.040	.237	.096
Fair	**.510**	−.010	.119	.225
Free-spirited	**.504**	.115	−.015	.210
Positive	.141	**.785**	−.041	−.106
Creative	.035	**.779**	−.170	.109
Determined	−.055	**.753**	.183	−.029
Flexible	.068	**.733**	−.020	−.126
Enthusiastic	.036	**.679**	.099	−.031
Receptive	.206	**.676**	−.157	−.022
Rational	−.059	**.670**	.115	.073
Deliberate	−.222	**.665**	.210	.139
Courageous	−.062	**.659**	.063	.080
Responsible	.028	.037	**.832**	−.061
Disciplined	.057	−.012	**.709**	.056
Patient	.133	.046	**.656**	.017
Reliable	.201	.120	**.541**	−.036
Analytical	.238	−.019	−.177	**.804**
Intelligent	.017	.076	.011	**.773**
Organized	−.100	−.013	.212	**.727**
% Variance explained	48.2%	7.9%	3.7%	2.7%

Note: Primary factor loadings are shown in bold.

**Fig 1:**
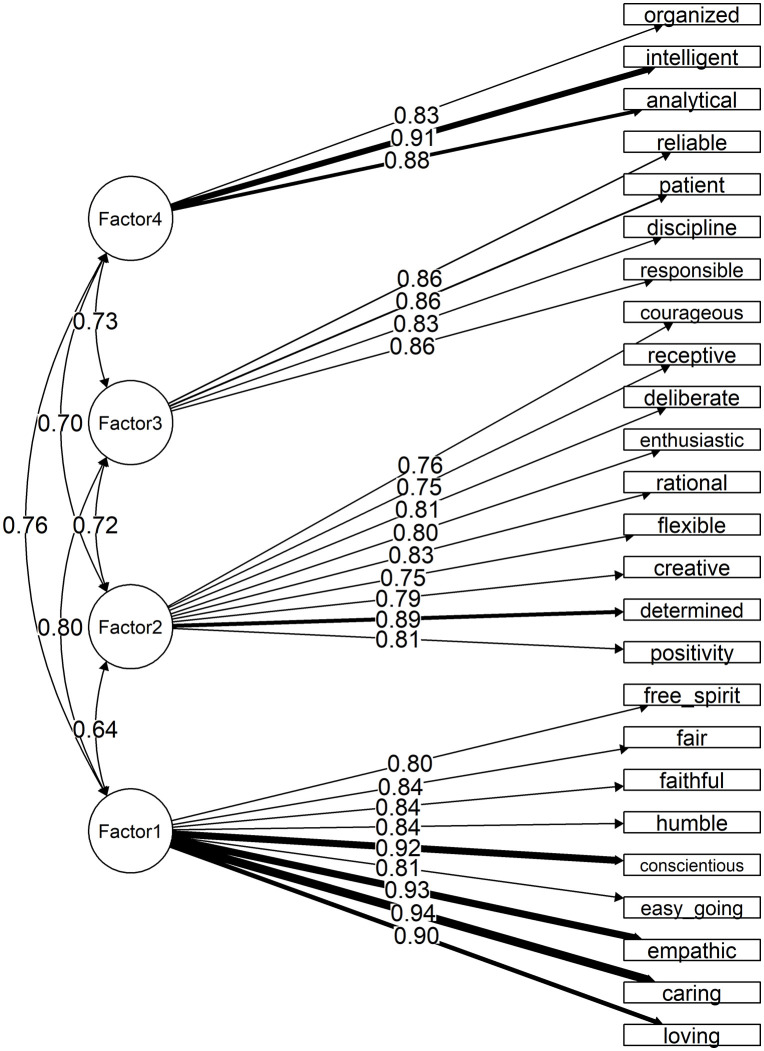
Results of the CFA of the final IRPS (25 items).

Based on the four-factor model, CFA indicated suboptimal model fit [χ² (269) = 949.56, *p* < .001; robust RMSEA [90%CI] =  .104 [.098,  .111]; robust CFI = .888; robust TLI = .876], but acceptable residual-based fit (SRMR = .045). Despite these limitations, the CFA supported the intended factor structure, with strong factor loadings ranging from  .75 to  .94 (Fig 1). Application of the Fornell-Larcker criterion demonstrated that the square root of AVEs of all factors was higher than the correlation between them (S11 Table), supporting the distinction among the four factors. Based on the theme shared among items within each factor, the domains were labeled as follows; Factor 1 “Compassionate and Ethical Nature”; Factor 2, “Adaptable Mindset”; Factor 3, “Responsible Spirit”; and Factor 4, “Logical Perspective”.

Given the high correlations among the four factors (.64−.80), a bifactor CFA model with one general factor and four orthogonal specific factors was tested to clarify the relative contribution of a general internal resources factor versus domain-specific factors. The bifactor model revealed that all items loaded substantially on the general factor (standardized loadings = .56–.81), indicating a strong overarching construct underlying the scale (S12 Table). Loadings on the specific factors were consistently smaller than those on the general factor and varied across domains (Compassionate and Ethical Nature:  .24–.65; Adaptable Mindset:  .47–.56; Responsible Spirit:  .29–.44; Logical Perspective:  .34–.55) (S12 Table). Although the bifactor model demonstrated a statistically significant improvement in fit compared with the correlated four-factor model (Δχ² = 106.07, Δdf = 19, p < .001), absolute model fit indices remained mixed ([χ² (250) = 835.60, *p* < .001; robust RMSEA [90%CI] =  .100 [.093,  .107]; robust CFI = .904; robust TLI = .885; SRMR = .040), indicating that neither model provided an optimal representation of the data.

### Relations to external measures

The medians and interquartile ranges of the IRPS subscales can be found in Table 3. Spearman’s correlations between the IRPS (25 items) and other measures are also presented in Table 3. The IRPS total score showed significant positive correlation with the RI-9 (ρ [95%CI] =  .58 [.52,  .65], *p* < .001), WHO-5 (ρ [95%CI] =  .38 [.30,  .46], *p* < .001), and RSES-TR (ρ [95%CI] =  .37 [.29,  .46], *p* < .001), which supported its convergent validity. Discriminant validity was examined through correlations with depression (PHQ-9) and anxiety (GAD-7) measures. The IRPS total score demonstrated statistically negative correlations with both the PHQ-9 (ρ [95%CI] = − .41 [−.49, −.34], *p* < .001), and the GAD-7 (ρ [95%CI] = − .34 [−.42, −.26], *p* < .001). The correlations between the four IRPS factors and external measures were also aligned with the full-scale IRPS (Table 3).

**Table 3:** Correlations between IRPS factors and other measures with 95% confidence intervals.

Measure	IRPS Factor 1	IRPS Factor 2	IRPS Factor 3	IRPS Factor 4	IRPS Total
**Median** (IQR)	42 (39–48)	34 (34–38)	15 (15–19)	9 (9–10)	101 (101–113)
**IRPS Total**	.87**	.84** [.80, .88]	.81** [.77, .84]	.76** [.71, .80]	1
**RI-9**	.51** [.44, .59]	.49** [.42, .56]	.50** [.42, .57]	.50** [.42, .57]	.58** [.52, .65]
**RSES-TR**	.29** [.20, .37]	.38** [.30, .46]	.29** [.21, .38]	.31** [.23, .40]	.37** [.29, .46]
**WHO-5**	.27** [.18, .35]	.46** [.39, .54]	.25** [.17, .34]	.25** [.16, .33]	.38** [.30, .46]
**PHQ-9**	–.33** [–.41, –.25]	–.39** [–.47, –.31]	–.33** [–.41, –.25]	–.37** [–.45, –.29]	–.41** [–.49, –.34]
**GAD-7**	–.25** [–.33, –.17]	–.33** [–.41, –.25]	–.26** [–.34, –.18]	–.31** [–.39, –.23]	–.34** [–.42, –.26]

Note. Values represent Spearman’s correlation coefficients, with 95% confidence intervals shown in brackets.

IRPS = Internal Resource Perception Scale; RI-9 = Resilience Inventory; RSES-TR = Rosenberg Self-Esteem Scale, Thai Revised; WHO-5 = WHO-5 Well-Being Index; PHQ-9 = Patient Health Questionnaire; GAD-7 = Generalized Anxiety Disorder Scale.

**p < .001.

### Reliability

The reliability analysis revealed excellent internal consistency of the 25-item IRPS (McDonald’s omega = .96, Cronbach’s alpha = .96). At the factor level, the internal consistency was good to excellent, indicating reliable item aggregation within each domain. The McDonald’s omega and Cronbach’s alpha coefficients were similar for each factor: Factor 1 (9 items) =  .94, Factor 2 (9 items) =  .92, Factor 3 (4 items) =  .87, and Factor 4 (3 items) =  .86. The item-total and item-rest correlations of all four factors were moderate to strong (range:  .64−.89, in S13 Table), supporting the reliability within each factor. For the bifactor model, the general factor accounted for a substantial proportion of reliable variance (ωₕ =  .85), whereas omega hierarchical values for the specific factors were comparatively lower (ωₛ range = .14–.36; S12 Table), suggesting limited unique reliable variance beyond the general factor.

## Discussion

Positive self-perception is crucial for maintaining optimal mental health. An individual’s internal resource perception not only affects their mental healing process but also facilitates personal growth. However, there has been a notable absence of a tool that focuses on measuring this perception. To bridge this gap, the authors developed a novel instrument and evaluated its psychometric properties.

The development of this tool included an extensive literature review and a focus group interview and also drew upon the researchers’ expertise in psychotherapy. Kendall’s coefficient of concordance of 45 items (W = .487) indicated a moderate level of agreement among the experts. While the level of agreement was not particularly high, it demonstrated reasonable consistency, given the subjective nature of the items and potential differences in the raters’ perspectives. Moderate agreement is common in assessments of complex constructs or multifaceted constructs, where some degree of variation is expected [67,68].

Out of the 45 initial items, 42 were validated using I-CVI and S-CVI calculations. The I-CVI of the IRPS ranged from  .80 to 1.00, and the overall S-CVI was  .94. Both values are higher than the recommended values proposed by Polit and Beck [69]. This indicates that each item is relevant to the internal resources of the individuals we aimed to measure.

After 17 items were deleted due to low factor loadings or high cross-loadings during the process of item reduction and factor extraction, the tool finally consisted of 25 items. The EFA revealed four factors: Compassionate and Ethical Nature, Adaptable Mindset, Responsible Spirit, and Logical Perspective. Although the CFA yielded global fit indices that did not meet all conventional cutoffs, it supported the intended factor structure, with all items loading significantly on their hypothesized factors, which are theoretically coherent and empirically distinguishable. However, the high correlations among factors suggested substantial shared variance. We therefore tested a bifactor model and found that this shared variance is largely accounted for by a general factor, on which all items loaded strongly. In contrast, loadings on the specific factors were attenuated once the general factor was included. These findings suggest that the total score represents the most psychometrically robust summary of the construct measured by the scale. Although the subscales retain conceptual relevance and capture domain-specific nuances, bifactor analyses indicate that their scores largely reflect variance shared with the general factor. From an applied perspective, these results support the use of the total IRPS score as the primary index of internal resource perception, with domain scores serving an auxiliary role for qualitative profiling or exploratory analyses.

To clarify the thematic content captured by the IRPS, we noticed that the Compassionate and Ethical Nature domain emerged as a particularly salient way individuals conceptualize their internal resources, which highlights the central role of moral and prosocial values in how individuals perceive themselves. This finding aligns with cross-cultural studies in positive psychology emphasizing the universality of virtues, such as love, concern for others, and fairness [70,71]. These resources have been associated with psychological well-being, social harmony, and resilience [72–74], indicating that individuals may perceive these qualities not only as socially desirable but also as deeply rooted internal strengths. The naming of this factor is theoretically grounded in research on compassion, empathy, and prosocial behavior and emphasizes moral values and concern for others as essential components of human flourishing [75,76]. By aligning closely with these established constructs, the label “Compassionate and Ethical Nature” reflects the convergence between the items in this domain and well-documented theories of prosocial motivation. This supports existing theory by showing that moral and prosocial traits are central internal resources perceived by individuals themselves.

The second domain, Adaptable Mindset, underlines the perceived value of cognitive and emotional flexibility and openness to change. This reflects a key principle of growth mindset theory and adaptive coping frameworks [77,78], and is supported by empirical work showing that adaptability facilitates effective coping, stress management, and post-traumatic growth [77,79,80]. The items that define this domain (e.g., positive, flexible, receptive, and courageous) correspond conceptually to psychological flexibility and a growth mindset, which highlight openness to new experiences and a willingness to adapt when circumstances change [77,81]. The presence of this domain in the IRPS suggests that perceiving these qualities enables individuals to navigate challenges and view change as an opportunity for growth rather than a threat. This extends the understanding of the growth mindset and adaptive coping frameworks by highlighting perceived internal flexibility as a measurable personal resource.

The third domain, Responsible Spirit, reflects internalized responsibility, self-discipline, and perseverance. These elements are conceptually close to conscientiousness [82–84] and have been shown to predict goal attainment, self-efficacy, and persistence in the face of obstacles [85,86]. The items in this domain (e.g., responsible, patient, disciplined, and reliable) map directly onto established definitions of conscientiousness and related constructs, which provide a theoretical foundation for the chosen label. Our results suggest that perceiving oneself as responsible supports a sense of self-efficacy and competence, especially in cultural contexts in which fulfilling roles and commitments is highly valued. This complements personal theory by demonstrating that the internal perception of conscientiousness-related traits, as reflected in the Responsible Spirit factor, contributes to adaptive functioning.

The fourth domain, Logical Perspective, brings attention to the rational and cognitive dimension of internal resources. While emotional and moral elements are often emphasized in discussions of psychological strengths, our findings underscore the importance of mental clarity, analytical thinking, and problem-solving abilities. The items that define this domain reflect constructs of critical thinking and cognitive reflection, which have been associated with executive functioning and rational decision-making in complex environments [87,88]. These cognitive capacities have been linked to executive functioning and adaptive functioning in complex environments and recognized as protective factors [89,90]. This supports cognitive theories of executive function by illustrating that individuals’ awareness of their logical skills constitutes a meaningful internal resource.

Although this study was conducted in Thailand, the four domains identified—Compassionate and Ethical Nature, Adaptable Mindset, Responsible Spirit, and Logical Perspective—reflect psychological qualities that are widely recognized across cultures [10,11]. The strong prominence of compassion and acceptance in the Thai data may reflect cultural values rooted in Buddhism and collectivism, which emphasize harmony and mindfulness [91,92]. At the same time, these qualities are consistent with universal frameworks in positive psychology and resilience research [12,93], suggesting that, while culturally grounded, the findings may also be relevant to broader populations. Together, these findings indicate that internal resource perception is a multifaceted construct, with each dimension reflecting a distinct thematic expression of psychological resilience, well-being, and adaptive functioning [94,95]. By integrating our results with existing theoretical and empirical work, the IRPS offers a novel and nuanced understanding of how individuals recognize and utilize their internal resources, complementing existing measures that focus solely on trait presence or behavior.

The internal consistency reliability of the IRPS was remarkably high, with McDonald’s omega and Cronbach’s alpha coefficients of  .96. Both coefficients for each factor also fell within a very good range, from  .86 to  .94. Additionally, the IRPS demonstrated good construct validity through its positive correlations with measures of resilience (RI-9; ρ = .58, *p* < .001), well-being (WHO-5; ρ = .38, *p* < .001), and self-esteem (RESE-TR; ρ = .37, *p* < .001); in addition, it had negative correlations with depression (PHQ-9; ρ = −.41, *p* < .001) and anxiety (GAD-7; ρ = −.34, *p* < .001). These associations ranged from weak to moderate in magnitude [96]. This also means that the higher IRPS scores were associated with greater life resilience, better quality of life, and higher self-esteem, while being associated with lower levels of depression and anxiety.

### Limitations

This study has some limitations. First, the study employed EFA, which is useful for identifying a potential factor structure, but did not confirm the stability of the factors in an independent sample. The internal structure analysis revealed that while Factor 1 explained 48.2% of the total variance, Factor 2–4 each accounted for less than 10%, raising questions about the extent to which these factors capture variance beyond the general construct. Moreover, Factor 4 contained only three items, which might yield unreliable estimates and limit content coverage. However, the CFA in the same sample confirmed strong factor loadings for all items on their intended factors under the four-factor structure despite suboptimal global fit indices. The Fornell-Larcker criterion also suggested that all factors are distinct, supporting their retention to provide a multidimensional evaluation of the internal resources. Although the four-factor structure was theoretically interpretable and empirically supported, bifactor analyses suggested that a substantial proportion of reliable variance is attributable to a general internal resources factor, with more limited unique variance in the specific domains. This suggests that the IRPS may primarily function as a unidimensional measure with meaningful subdomains that capture stylistic or thematic expressions of internal resource perception. Future studies should replicate these findings in independent samples and further examine the incremental and predictive validity of subscale scores beyond the total score.

Second, the test−retest reliability of the IRPS was not assessed in this study. This might restrict conclusions about the scale’s temporal stability. However, the authors plan to evaluate this test−retest reliability in a subsequent phase of the research.

Third, although the IRPS may resonate across cultural contexts because it encompasses universal human qualities−compassion, adaptability, responsibility, and reasoning− it was initially developed in Thailand. Thai people often cope with hardship through a culturally rooted form of acceptance that emphasizes letting go, finding more inner peace, and moving forward without or with less conflict [97]. This reflects the deep values of harmony, mindfulness, and compassion within Thai culture. Therefore, further translation and cultural adaptation may be required for the cross-cultural use of this tool.

Fourth, convenience sampling was used to efficiently recruit participants from the general population. However, this approach may still be limited because participants were recruited based on accessibility and a willingness to participate; thus, there is a risk of selection bias, which should be considered when interpreting the results.

Lastly, individuals can be influenced by social desirability, memory biases, and limited self-awareness or understanding of each internal resource, which is common for self-administered tools [98]. However, this perception could reflect their mental health and well-being as mentioned above. Despite these limitations, the results of our study demonstrated excellent reliability and validity, indicating that the IRPS has the potential to be a valuable tool. Future research could further explore the stability of the IRPS score over time through longitudinal studies and examine the relationships between IRPS scores and other psychological constructs, such as coping styles and social support.

## Conclusions

The IRPS is the first tool developed to assess an individual’s subjective perception of internal resources and has demonstrated good validity and reliability. It can be used to evaluate individuals’ perceptions of internal resources, which may reflect their mental health. This tool has the potential to foster deeper self-understanding, learning, and personal growth, which may support better emotional regulation and stress management. While this tool demonstrated good psychometric properties, future research is required to explore and refine the tool’s potential in various settings.

## Supporting information

S1 Table Item Origins and Evidence Sources for the Initial Internal Resource Perception Scale (IRPS). (DOCX)

S2 Table I-CVI, descriptive statistics, and factor loadings of the 42-item IRPS. (DOCX)

S3 Table Parallel analysis and minimum average partial (MAP) test of 42-item IRPS. (DOCX)

S4 Table Model fitting indices of the 42-item IRPS. (DOCX)

S5 Table Parallel analysis and MAP test of 27-item IRPS. (DOCX)

S6 Table Factor loadings of the 27-item IRPS derived from the second round of EFA. (DOCX)

S7 Table Model fitting indices of the 27-item IRPS. (DOCX)

S8 Table Parallel analysis and MAP test of 25-item IRPS. (DOCX)

S9 Table Factor loadings of the 25-item IRPS derived from the third round of EFA. (DOCX)

S10 Table Model fitting indices of the 25-item IRPS. (DOCX)

S11 Table Factor correlations and Fornell-Larcker Criterion. (DOCX)

S12 Table Factor loadings of the 25-item IRPS based on the bifactor model. (DOCX)

S13 Table Item reliability statistics within each factor of the 25-item IRPS. (DOCX)

S1 Dataset Expert rated. (XLS)

S2 Dataset PASW statistic data editor. (SAV)

S1 File Internal Resource Perception Scale. (DOCX)
