# Supplementary material for: The development of the Internal Resource Perception Scale: Validity and reliability
Source: PLoS One. 2026 Apr 29;21(4):e0348075. doi: 10.1371/journal.pone.0348075 (PMC13127970; doi:10.1371/journal.pone.0348075)
Supplement: S1 Table — (DOCX) [file pone.0348075.s001.docx]

**S1 Table. Item Origins and Evidence Sources for the Initial Internal Resource Perception Scale (IRPS)**

| **Items** | **References (article)** | **References (websites)** | **Focus group** | **Clinical experience** |
| --- | --- | --- | --- | --- |
| 1. compassionate | -Peterson & Seligman (2004)  -Batson (2011)  -Gomori & Adaskin (2008)  -Satir (1972) | [viacharacter.org](https://www.viacharacter.org/www/character-strengths) | + | + |
| 2. receptive | -Peterson & Seligman (2004)  -Martin & Rubin (1995) | [viacharacter.org](https://www.viacharacter.org/www/character-strengths) |  | + |
| 3. disclose | -Jourard (1971)  -Pennebaker (1997) |  | + | + |
| 4. flexible | -Hayes et al. (2006)  -Bond&Hayes (2006) | [apa.org](https://www.apa.org/topics/resilience) | + | + |
| 5. sincere | -Kernis & Goldman (2006) |  | + | + |
| 6. enthusiastic | -Ryan & Frederick (1997)  -Peterson & Seligman (2004) | [positivepsychology.com](https://positivepsychology.com/engagement/)  onestopforwriters.com /positive traits | + | + |
| 7. lively | -Fredrickson (2001)  -Peterson & Seligman (2004) |  | + | + |
| 8. creative | -Runco & Jaeger (2012)  -Peterson & Seligman (2004)  -Satir, Banmen, Gerber, Gomori (1991) | [viacharacter.org](https://www.viacharacter.org/www/character-strengths)  onestopforwriters.com /positive traits | + | + |
| 9. curious | -Kashdan et al. (2004)  -Peterson & Seligman (2004) | [viacharacter.org](https://www.viacharacter.org/www/character-strengths)  onestopforwriters.com /positive traits | + | + |
| 10. determined | -Baumeister & Vohs (2007)  -Csikszentmihalyi (2004)  -Baumeister (2007) | onestopforwriters.com /positive traits | + | + |
| 11. positive | -Carver & Scheier (2014)  -Peterson & Seligman (2004) | [positivepsychology.com](https://positivepsychology.com/optimism/) | + | + |
| 12. courageous | -Peterson & Seligman (2004)  -Gomori & Adaskin (2008) | [viacharacter.org](https://www.viacharacter.org/www/character-strengths)  onestopforwriters.com /positive traits | + | + |
| 13. deliberate | -Kabat-Zinn (1994)  -Peterson & Seligman (2004)  -Costa&McCrae(1992) | [positivepsychology.com](https://positivepsychology.com/mindfulness/) | + | + |
| 14. honest | -Peterson & Seligman (2004)  -Satir (1972) | [viacharacter.org](https://www.viacharacter.org/www/character-strengths)  onestopforwriters.com /positive traits | + | + |
| 15. rational | -Halpern (2014) | [simplypsychology.org](https://www.simplypsychology.org/cognition.html) | + | + |
| 16. responsible | -Eisenberg et al. (2006)  -Kinena(2014)  -Satir (1972) | [verywellmind.com](https://www.verywellmind.com/the-big-five-personality-dimensions-2795422)  onestopforwriters.com /positive traits | + | + |
| 17. reliable | -Costa & McCrae (1992) | [verywellmind.com](https://www.verywellmind.com/the-big-five-personality-dimensions-2795422) |  | + |
| 18. frugal | -Peterson & Seligman (2004)  -Lastovicka(1999) |  | + | + |
| 19. patient | -Schnitker (2012) | [positivepsychology.com](https://positivepsychology.com/mindfulness/) [apa.org](https://www.apa.org/topics/resilience) | + | + |
| 20. strong | -Masten (2001) |  | + | + |
| 21. discipline | -Duckworth & Seligman (2005)  -Peterson & Seligman (2004) | [simplypsychology.org](https://www.simplypsychology.org/self-control.html)  onestopforwriters.com /positive traits | + | + |
| 22. gratitude | -Emmons & McCullough (2003)  -Peterson & Seligman (2004) | [viacharacter.org](https://www.viacharacter.org/www/character-strengths) | + | + |
| 23. emotionally stable | -Goleman (1995)  -Gross (2015)  -Peterson & Seligman (2004) | onestopforwriters.com /positive traits |  | + |
| 24. calm | -Kabat-Zinn (1994) | onestopforwriters.com /positive traits | + | + |
| 25. gentle | -Digman (1990) | onestopforwriters.com /positive traits | + | + |
| 26. sensitive | -Satir, Banmen, Gerber, Gomori (1991) | spines.com/positive-  character-trait  onestopforwriters.com /positive traits |  | + |
| 27. self-aware | -Duval & Wicklund (1972) |  |  | + |
| 28. humoring | -Martin (2007)  -Peterson & Seligman (2004)  -Gomori & Adaskin (2008)  -Satir, Banmen, Gerber, Gomori (1991) | [viacharacter.org](https://www.viacharacter.org/www/character-strengths) | + | + |
| 29. romantic | -Hazan & Shaver (1987) |  | + |  |
| 30. challenge-loving | -Dweck (2006) |  |  | + |
| 31. organized | -Costa & McCrae (1992) | [mindtools.com](https://www.mindtools.com/pages/main/newMN_HTE.htm)  onestopforwriters.com /positive traits | + | + |
| 32. fair | -Peterson & Seligman (2004) | [viacharacter.org](https://www.viacharacter.org/www/character-strengths) | + | + |
| 33. intelligent | -Ardelt (2004)  -Peterson & Seligman (2004)  -Gomori & Adaskin (2008)  -Satir, Banmen, Gerber, Gomori (1991) | [simplypsychology.org](https://www.simplypsychology.org/intelligence.html)  onestopforwriters.com /positive traits | + | + |
| 34. independent | -Deci & Ryan (2000) | onestopforwriters.com /positive traits | + | + |
| 35. humble | -Tangney (2000)  -Peterson & Seligman (2004) | [viacharacter.org](https://www.viacharacter.org/www/character-strengths)  onestopforwriters.com /positive traits | + | + |
| 36. analytical | -Stanovich & West (2000)  -Clifton&Nelson(1992) | [criticalthinking.org](https://www.criticalthinking.org/pages/defining-critical-thinking/766)  onestopforwriters.com /positive traits | + | + |
| 37. faithful | -Peterson & Seligman (2004) | [viacharacter.org](https://www.viacharacter.org/www/character-strengths) | + | + |
| 38. free-spirited | -Deci & Ryan (2000) | [positivepsychology.com](https://positivepsychology.com/autonomy/) |  | + |
| 39. holistic | -Baumeister & Leary (1995) |  |  | + |
| 40. easy-going |  |  | + | + |
| 41. loving | -Lee (1973)  -Fehr (1994)  -Peterson & Seligman (2004)  -Gomori & Adaskin (2008)  -Satir (1972) | [positivepsychology.com](https://positivepsychology.com/love/) | + | + |
| 42. caring | -Davis (1994)  -Batson (2011)  -Satir, Banmen, Gerber, Gomori (1991) | [viacharacter.org](https://www.viacharacter.org/www/character-strengths) | + | + |
| 43. self-assured | -Bandura (1997) |  |  | + |
| 44. conscientious | -McCrae & Costa (1997)  -Goldberg(1990) | [verywellmind.com](https://www.verywellmind.com/the-big-five-personality-dimensions-2795422) | + | + |
| 45. empathetic | -Davis (1994)  -Peterson & Seligman (2004) | [mindtools.com](https://www.mindtools.com/pages/article/newCDV_59.htm)  onestopforwriters.com /positive traits | + | + |
