# Supplementary material for: The development of the Internal Resource Perception Scale: Validity and reliability
Source: PLoS One. 2026 Apr 29;21(4):e0348075. doi: 10.1371/journal.pone.0348075 (PMC13127970; doi:10.1371/journal.pone.0348075)
Supplement: S2 Table — (DOCX) [file pone.0348075.s002.docx]

**S2 Table. I-CVI, descriptive statistics, and factor loadings of the 42-item IRPS**

| **Resources**  **“I am…”** | I-CVI | Median | IQR | Mean | SD | Factor loadings | | | | | | | | |
| --- | --- | --- | --- | --- | --- | --- | --- | --- | --- | --- | --- | --- | --- | --- |
|  |  |  |  |  |  | 5-factor model (MAP test) | | | | | 4-factor model (parallel analysis) | | | |
|  |  |  |  |  |  | 1 | 2 | 3 | 4 | 5 | 1 | 2 | 3 | 4 |
| loving | 1.0 | 3 | 3-4 | 3.32 | 0.74 | **.866** | -.017 | -.120 | .003 | .157 | -.021 | **.869** | -.143 | .158 |
| caring | 1.0 | 3 | 3-4 | 3.38 | 0.71 | **.859** | -.024 | .020 | .008 | .061 | -.033 | **.861** | .014 | .072 |
| empathetic | 1.0 | 3 | 3-4 | 3.40 | 0.70 | **.846** | .001 | .036 | .018 | -.006 | -.011 | **.847** | .041 | .018 |
| conscientious | 0.8 | 3 | 3-4 | 3.36 | 0.72 | **.764** | -.020 | .094 | .116 | -.061 | -.032 | **.760** | .120 | .051 |
| easy-going | 1.0 | 3 | 3-4 | 3.34 | 0.70 | **.753** | .013 | -.199 | .053 | .261 | .017 | **.755** | -.229 | .292 |
| humble | 1.0 | 3 | 3-4 | 3.31 | 0.73 | **.655** | -.026 | .199 | .032 | -.024 | -.037 | **.654** | .213 | .007 |
| fair | 1.0 | 3 | 3-4 | 3.29 | 0.69 | **.572** | .074 | .074 | .248 | -.144 | .066 | **.563** | .129 | .078 |
| faithful | 1.0 | 3 | 3-4 | 3.17 | 0.85 | **.529** | -.016 | .167 | .091 | .121 | -.019 | **.528** | .170 | .182 |
| free-spirited | 0.8 | 3 | 3-4 | 3.21 | 0.79 | **.469** | .030 | -.017 | .266 | .167 | .035 | **.470** | -.007 | .374^a^ |
| gratitude | 1.0 | 4 | 3-4 | 3.49 | 0.68 | **.431** | .038 | .425^a^ | -.051 | -.005 | .027 | **.425** | .435^a^ | -.055 |
| independent | 1.0 | 3 | 3-4 | 3.31 | 0.70 | **.418** | -.012 | -.013 | .382^a^ | .147 | -.005 | **.417** | .016 | .448^a^ |
| gentle | 1.0 | 3 | 3-4 | 3.14 | 0.79 | **.415** | .031 | .338^a^ | -.136 | .193 | .031 | **.413** | .303^a^ | .054 |
| sensitive | 1.0 | 3 | 3-4 | 3.09 | 0.85 | **.266^b^** | .025 | .257 | -.020 | .068 | .022 | **.264**^b^ | .257^a^ | .036 |
| sincere | 1.0 | 3 | 3-4 | 3.36 | 0.68 | .312^a^ | **.799** | -.054 | -.152 | -.189 | **.794** | .312^a^ | -.037 | -.321^a^ |
| determined | 1.0 | 3 | 3-4 | 3.13 | 0.75 | -.088 | **.759** | .129 | .086 | -.009 | **.769** | -.090 | .155 | .025 |
| honest | 1.0 | 3 | 3-4 | 3.33 | 0.76 | .213 | **.719** | .103 | -.113 | -.233 | **.711** | .210 | .133 | -.328^a^ |
| creative | 1.0 | 3 | 2-4 | 2.90 | 0.86 | -.069 | **.713** | -.154 | .127 | .274 | **.738** | -.065 | -.169 | .320^a^ |
| rational | 1.0 | 3 | 3-4 | 3.20 | 0.76 | -.058 | **.695** | .099 | .198 | -.153 | **.693** | -.056 | .153 | -.004 |
| positive | 1.0 | 3 | 3-4 | 3.10 | 0.81 | .043 | **.693** | .014 | -.054 | .179 | **.707** | .047 | -.007 | .087 |
| curious | 1.0 | 3 | 2-4 | 2.86 | 0.84 | -.172 | **.680** | -.209 | .312^a^ | .146 | **.704** | -.168 | -.187 | .358^a^ |
| lively | 1.0 | 3 | 3-3 | 2.95 | 0.84 | .005 | **.677** | .024 | -.235 | .455^a^ | **.678** | .016 | -.042 | .180 |
| enthusiastic | 1.0 | 3 | 3-3 | 2.93 | 0.80 | -.007 | **.671** | .021 | .050 | .108 | **.685** | -.007 | .023 | .107 |
| deliberate | 1.0 | 3 | 2-3 | 2.90 | 0.87 | -.231 | **.642** | .214 | .246 | -.063 | **.648** | -.228 | .264 | .108 |
| flexible | 0.8 | 3 | 3-4 | 2.95 | 0.76 | -.023 | **.610** | .032 | -.073 | .199 | **.624** | -.020 | .005 | .091 |
| receptive | 1.0 | 3 | 3-3 | 3.04 | 0.75 | .144 | **.598** | -.129 | .018 | .138 | **.612** | .149 | -.143 | .118 |
| compassionate | 1.0 | 3 | 3-4 | 3.23 | 0.65 | .364^a^ | **.582** | -.007 | -.248 | .028 | **.580** | .366^a^ | -.034 | -.196 |
| courageous | 1.0 | 3 | 2-3 | 2.89 | 0.88 | -.122 | **.580** | .050 | .188 | .155 | **.598** | -.123 | .060 | .267 |
| discipline | 0.8 | 3 | 3-4 | 3.08 | 0.82 | .020 | -.053 | **.754** | .062 | .071 | -.058 | .010 | **.778** | .092 |
| patient | 1.0 | 3 | 3-4 | 3.24 | 0.82 | .070 | .007 | **.713** | .042 | .052 | .002 | .061 | **.735** | .058 |
| responsible | 0.8 | 3 | 3-4 | 3.31 | 0.73 | .082 | .050 | **.696** | .037 | -.020 | .042 | .071 | **.729** | -.011 |
| strong | 0.8 | 3 | 3-4 | 3.22 | 0.80 | .019 | -.041 | **.692** | -.037 | .340^a^ | -.030 | .021 | **.649** | .255 |
| emotionally-stable | 1.0 | 3 | 2-4 | 2.92 | 0.90 | -.126 | .035 | **.597** | .038 | .441^a^ | .058 | -.116 | **.546** | .394^a^ |
| calm | 0.8 | 3 | 3-4 | 2.98 | 0.85 | .028 | .015 | **.510** | -.049 | .443^a^ | .036 | .038 | **.446** | .329^a^ |
| reliable | 0.8 | 3 | 3-4 | 3.37 | 0.67 | .221 | .117 | **.505** | .037 | -.046 | .109 | .213 | **.535** | -.028 |
| self-aware | 1.0 | 3 | 3-4 | 3.15 | 0.73 | .232^a^ | .010 | **.372^b^** | .053 | .158 | .011 | .228^a^ | **.372^b^** | .173 |
| analytical | 1.0 | 3 | 3-4 | 3.07 | 0.77 | .297 | .021 | -.104 | **.712** | -.038 | .038 | .292 | .009 | **.513** |
| intelligent | 1.0 | 3 | 3-3 | 2.98 | 0.73 | .045 | .073 | .070 | **.705** | .074 | .095 | .055 | .156 | **.602** |
| organized | 0.8 | 3 | 3-3 | 2.93 | 0.82 | .000 | .078 | .214 | **.646** | -.095 | .093 | .014 | .303^a^ | **.405** |
| self-assured | 0.8 | 3 | 3-4 | 3.04 | 0.88 | .252^a^ | -.098 | .105 | **.411** | .226 | -.088 | .251 | .127 | **.543** |
| humoring | 1.0 | 3 | 3-4 | 3.10 | 0.80 | .271^a^ | .084 | .067 | -.019 | **.455** | .102 | .272^a^ | .016 | **.377^b^** |
| challenge-loving | 0.8 | 3 | 2-3 | 2.86 | 0.89 | .051 | .149 | .158 | .142 | **.451** | .171 | .055 | .124 | **.501** |
| holistic | 1.0 | 3 | 3-4 | 3.05 | 0.80 | .431^a^ | -.007 | .033 | .059 | **.436** | .009 | .431^a^ | -.009 | **.433** |
| % variance explained |  |  |  |  |  | 45.7% | 6.4% | 3.6% | 2.8% | 2.3% | 45.7% | 6.4% | 3.5% | 2.7% |

Note: Primary factor loadings are shown in bold.

^a^ Alternative absolute factor loading > 0.3 or a difference between the primary and alternative loadings > 0.2

^b^ Primary factor loading < 0.4
