# Supplementary material for: The development of the Internal Resource Perception Scale: Validity and reliability
Source: PLoS One. 2026 Apr 29;21(4):e0348075. doi: 10.1371/journal.pone.0348075 (PMC13127970; doi:10.1371/journal.pone.0348075)
Supplement: S3 Table — (DOCX) [file pone.0348075.s003.docx]

**S3 Table. Parallel analysis and minimum average partial (MAP) test of 42-item IRPS**

| Factor | Parallel Analysis | | MAP test | |
| --- | --- | --- | --- | --- |
|  | Raw Data Eigenvalues | 95th Percentile of Random Data Eigenvalues | Average squared partial correlation | Average 4^th^ power partial correlation |
| 0 |  |  | 0.2128 | 0.054 |
| 1 | 19.58859 | 1.6556 | 0.0264 | 0.002 |
| 2 | 3.080104 | 1.573269 | 0.0167 | 0.0009 |
| 3 | 1.863906 | 1.51751 | 0.0135 | 0.0006 |
| 4 | **1.541507** | **1.472811** | 0.0117 | 0.0004 |
| 5 | 1.328178 | 1.429631 | **0.0104** | **0.0003** |
| 6 | 1.102133 | 1.393763 | 0.0106 | 0.0003 |
| 7 | 0.906503 | 1.360915 | 0.0108 | 0.0004 |
| 8 | 0.795457 | 1.33023 | 0.0115 | 0.0004 |
| 9 | 0.733473 | 1.299137 | 0.012 | 0.0005 |
| 10 | 0.654152 | 1.27039 | 0.0129 | 0.0006 |
| 11 | 0.644004 | 1.239994 | 0.0137 | 0.0007 |
| 12 | 0.580086 | 1.215253 | 0.0147 | 0.0009 |
| 13 | 0.550398 | 1.189877 | 0.0158 | 0.001 |
| 14 | 0.514219 | 1.164063 | 0.0169 | 0.0012 |
| 15 | 0.494617 | 1.14096 | 0.018 | 0.0014 |
| 16 | 0.482162 | 1.118182 | 0.0196 | 0.0016 |
| 17 | 0.452911 | 1.09515 | 0.0211 | 0.0019 |
| 18 | 0.432561 | 1.072375 | 0.0231 | 0.0021 |
| 19 | 0.40917 | 1.04982 | 0.0251 | 0.0024 |
| 20 | 0.406158 | 1.028663 | 0.0275 | 0.0028 |
| 21 | 0.380707 | 1.005558 | 0.0299 | 0.0032 |
| 22 | 0.360836 | 0.984097 | 0.0325 | 0.0038 |
| 23 | 0.346673 | 0.962238 | 0.0357 | 0.0046 |
| 24 | 0.341884 | 0.942542 | 0.0392 | 0.0055 |
| 25 | 0.324453 | 0.922418 | 0.0428 | 0.006 |
| 26 | 0.300426 | 0.902383 | 0.0469 | 0.0074 |
| 27 | 0.29392 | 0.881164 | 0.0518 | 0.0087 |
| 28 | 0.282215 | 0.862501 | 0.0577 | 0.0103 |
| 29 | 0.26753 | 0.844277 | 0.0642 | 0.0123 |
| 30 | 0.253576 | 0.823494 | 0.0712 | 0.0154 |
| 31 | 0.248643 | 0.804365 | 0.0793 | 0.019 |
| 32 | 0.236807 | 0.783733 | 0.091 | 0.024 |
| 33 | 0.22616 | 0.764746 | 0.1043 | 0.0316 |
| 34 | 0.22197 | 0.745962 | 0.1198 | 0.0384 |
| 35 | 0.211752 | 0.725896 | 0.1467 | 0.0535 |
| 36 | 0.199165 | 0.704135 | 0.1685 | 0.0692 |
| 37 | 0.187343 | 0.684005 | 0.2125 | 0.1005 |
| 38 | 0.179736 | 0.664652 | 0.2773 | 0.1515 |
| 39 | 0.167046 | 0.641872 | 0.3555 | 0.2261 |
| 40 | 0.147783 | 0.619461 | 0.492 | 0.371 |
| 41 | 0.136994 | 0.596233 | 1 | 1 |
| 42 | 0.124092 | 0.567739 |  |  |

Note: The number of factors to be extracted based on each method is shown in bold.
