# Supplementary material for: The development of the Internal Resource Perception Scale: Validity and reliability
Source: PLoS One. 2026 Apr 29;21(4):e0348075. doi: 10.1371/journal.pone.0348075 (PMC13127970; doi:10.1371/journal.pone.0348075)
Supplement: S4 Table — (DOCX) [file pone.0348075.s004.docx]

**S4 Table. Model fitting indices of the 42-item IRPS**

| Model | χ² p-value | RMSEA [90%CI] | CFI | TLI | SRMR | BIC | % variance explained |
| --- | --- | --- | --- | --- | --- | --- | --- |
| **5-factor** | **< .001** | **.084 [.082, .087]** | **.825** | **.814** | **.058** | **37116** | **60.8** |
| 4-factor | < .001 | .086 [.083, .089] | .817 | .806 | .060 | 37229 | 58.3 |
| 3-factor | < .001 | .089 [.087, .092] | .801 | .790 | .061 | 37479 | 55.5 |
| 2-factor | < .001 | .096 [.094, .099] | .769 | .757 | .062 | 38010 | 51.8 |
| 1-factor | < .001 | .114 [.112, .117] | .674 | .657 | .083 | 39608 | 45.4 |

Note: The best-fit model is shown in bold.
