# Supplementary material for: The development of the Internal Resource Perception Scale: Validity and reliability
Source: PLoS One. 2026 Apr 29;21(4):e0348075. doi: 10.1371/journal.pone.0348075 (PMC13127970; doi:10.1371/journal.pone.0348075)
Supplement: S5 Table — (DOCX) [file pone.0348075.s005.docx]

**S5 Table. Parallel analysis and MAP test of 27-item IRPS**

| Factor | Parallel Analysis | | MAP test | |
| --- | --- | --- | --- | --- |
|  | Raw Data Eigenvalues | 95th Percentile of Random Data Eigenvalues | Average squared partial correlation | Average 4^th^ power partial correlation |
| 0 |  |  | 0.229 | 0.0627 |
| 1 | 13.21842 | 1.511849 | 0.0342 | 0.003 |
| 2 | **2.442668** | **1.428579** | 0.0162 | 0.0008 |
| 3 | 1.286371 | 1.373919 | 0.015 | 0.0008 |
| 4 | 1.127139 | 1.32821 | **0.0138** | **0.0006** |
| 5 | 0.835851 | 1.280467 | 0.0149 | 0.0008 |
| 6 | 0.74111 | 1.243459 | 0.0166 | 0.0011 |
| 7 | 0.619206 | 1.210966 | 0.0188 | 0.0015 |
| 8 | 0.542859 | 1.176973 | 0.0217 | 0.0023 |
| 9 | 0.531731 | 1.147227 | 0.0243 | 0.0028 |
| 10 | 0.497644 | 1.115569 | 0.0281 | 0.0042 |
| 11 | 0.466946 | 1.087213 | 0.034 | 0.0059 |
| 12 | 0.452662 | 1.062168 | 0.0382 | 0.007 |
| 13 | 0.41309 | 1.033849 | 0.045 | 0.0094 |
| 14 | 0.380743 | 1.005447 | 0.0517 | 0.011 |
| 15 | 0.362284 | 0.983024 | 0.0601 | 0.0154 |
| 16 | 0.357149 | 0.954281 | 0.0687 | 0.0175 |
| 17 | 0.341229 | 0.931527 | 0.0782 | 0.0203 |
| 18 | 0.313037 | 0.907684 | 0.0919 | 0.025 |
| 19 | 0.305144 | 0.881358 | 0.1111 | 0.0332 |
| 20 | 0.280595 | 0.855333 | 0.1346 | 0.0448 |
| 21 | 0.253907 | 0.832368 | 0.1634 | 0.0639 |
| 22 | 0.251446 | 0.807904 | 0.2117 | 0.0997 |
| 23 | 0.241205 | 0.782348 | 0.2778 | 0.162 |
| 24 | 0.226669 | 0.756701 | 0.3994 | 0.2766 |
| 25 | 0.214681 | 0.727333 | 0.4874 | 0.3722 |
| 26 | 0.156 | 0.696868 | 1 | 1 |
| 27 | 0.140219 | 0.662935 |  |  |

Note: The number of factors to be extracted based on each method is shown in bold.
