# Supplementary material for: The development of the Internal Resource Perception Scale: Validity and reliability
Source: PLoS One. 2026 Apr 29;21(4):e0348075. doi: 10.1371/journal.pone.0348075 (PMC13127970; doi:10.1371/journal.pone.0348075)
Supplement: S6 Table — (DOCX) [file pone.0348075.s006.docx]

**S6 Table. Factor loadings of the 27-item IRPS derived from the second round of EFA**

| **Resources**  **“I am…”** |  | Factor loadings | | | | | |
| --- | --- | --- | --- | --- | --- | --- | --- |
|  |  | 4-factor model (MAP test) | | | | 2-factor model (parallel analysis) | |
|  |  | 1 | 2 | 3 | 4 | 1 | 2 |
| loving |  | **.943** | .045 | -.151 | -.021 | **.881** | -.111 |
| caring |  | **.897** | -.007 | .054 | -.061 | **.940** | -.109 |
| empathetic |  | **.849** | -.027 | .127 | -.072 | **.930** | -.106 |
| easy-going |  | **.819** | .098 | -.236 | .078 | **.751** | -.036 |
| conscientious |  | **.755** | -.053 | .215 | -.021 | **.907** | -.073 |
| humble |  | **.654** | -.056 | .232 | -.006 | **.816** | -.052 |
| faithful |  | **.538** | -.049 | .228 | .120 | **.749** | .019 |
| fair |  | **.524** | .016 | .157 | .149 | **.706** | .071 |
| free-spirited |  | **.505** | .095 | -.019 | .239 | **.623** | .130 |
| positive |  | .124 | **.796** | -.066 | -.083 | .027 | **.730** |
| determined |  | -.073 | **.774** | .167 | -.017 | -.032 | **.851** |
| creative |  | .014 | **.760** | -.203 | .184 | -.041 | **.765** |
| flexible |  | .047 | **.715** | -.057 | -.059 | -.037 | **.674** |
| rational |  | -.065 | **.713** | .136 | .009 | -.026 | **.790** |
| enthusiastic |  | .014 | **.676** | .061 | .034 | .030 | **.726** |
| deliberate |  | -.230 | **.670** | .211 | .139 | -.101 | **.846** |
| honest |  | .161 | **.668** | .207 | -.302^a^ | .123 | **.600** |
| receptive |  | .191 | **.667** | -.179 | .017 | .087 | **.592** |
| courageous |  | -.084 | **.662** | .032 | .140 | -.042 | **.756** |
| responsible |  | .020 | .033 | **.801** | -.010 | **.437** | .317^a^ |
| discipline |  | .047 | -.029 | **.678** | .126 | **.462** | .266^a^ |
| patient |  | .124 | .033 | **.626** | .078 | **.493** | .281 |
| reliable |  | .194 | .126 | **.530** | -.019 | **.472** | .291^a^ |
| intelligent |  | .013 | .033 | .040 | **.816** | .391^a^ | **.372^b^** |
| analytical |  | .264 | -.007 | -.083 | **.678** | **.521** | .215 |
| organized |  | -.077 | -.001 | .268 | **.653** | .351^a^ | **.365^b^** |
| Challenge-loving |  | .154 | .254 | .033 | **.318^b^** | .309^a^ | **.383^b^** |
| % variance explained |  | 47.6% | 7.7% | 3.4% | 2.8% | 47.3% | 7.6% |

Note: Primary factor loadings are shown in bold.

^a^ Alternative absolute factor loading > 0.3 or a difference between the primary and alternative loadings >

0.2

^b^ Primary factor loading < 0.4
