# Supplementary material for: The development of the Internal Resource Perception Scale: Validity and reliability
Source: PLoS One. 2026 Apr 29;21(4):e0348075. doi: 10.1371/journal.pone.0348075 (PMC13127970; doi:10.1371/journal.pone.0348075)
Supplement: S7 Table — (DOCX) [file pone.0348075.s007.docx]

**S7 Table. Model fitting indices of the 27-item IRPS**

| Model | χ² p-value | RMSEA [90% CI] | CFI | TLI | SRMR | BIC | % variance explained |
| --- | --- | --- | --- | --- | --- | --- | --- |
| **4-factor** | **< .001** | **.076 [.071, .080]** | **.907** | **.898** | **.051** | **23524** | **61.5** |
| 3-factor | < .001 | .084 [.080, .089] | .884 | .873 | .051 | 23744 | 58.3 |
| 2-factor | < .001 | .101 [.097, .105] | .831 | .817 | .078 | 24259 | 54.9 |
| 1-factor | < .001 | .130 [.125, .134] | .722 | .699 | .088 | 25349 | 47.0 |

Note: The best-fit model is shown in bold.
