# Supplementary material for: The development of the Internal Resource Perception Scale: Validity and reliability
Source: PLoS One. 2026 Apr 29;21(4):e0348075. doi: 10.1371/journal.pone.0348075 (PMC13127970; doi:10.1371/journal.pone.0348075)
Supplement: S8 Table — (DOCX) [file pone.0348075.s008.docx]

**S8 Table. Parallel analysis and MAP test of 25-item IRPS**

| Factor | Parallel Analysis | | MAP test | |
| --- | --- | --- | --- | --- |
|  | Raw Data Eigenvalues | 95th Percentile of Random Data Eigenvalues | Average squared partial correlation | Average 4^th^ power partial correlation |
| 0 |  |  | 0.2336 | 0.0657 |
| 1 | 12.38223 | 1.484749 | 0.0368 | 0.0033 |
| 2 | **2.38352** | **1.403229** | 0.0166 | 0.0008 |
| 3 | 1.268511 | 1.347254 | 0.0149 | 0.0008 |
| 4 | 1.040816 | 1.299554 | **0.0143** | **0.0008** |
| 5 | 0.732236 | 1.261336 | 0.0162 | 0.0009 |
| 6 | 0.614217 | 1.22243 | 0.0187 | 0.0013 |
| 7 | 0.544894 | 1.186177 | 0.0225 | 0.0023 |
| 8 | 0.541437 | 1.15278 | 0.0257 | 0.003 |
| 9 | 0.519956 | 1.123882 | 0.0293 | 0.0039 |
| 10 | 0.487317 | 1.094563 | 0.0342 | 0.0068 |
| 11 | 0.463415 | 1.065031 | 0.0407 | 0.0073 |
| 12 | 0.441008 | 1.036805 | 0.0481 | 0.0098 |
| 13 | 0.397416 | 1.007267 | 0.057 | 0.0142 |
| 14 | 0.363653 | 0.982198 | 0.0661 | 0.0192 |
| 15 | 0.357081 | 0.95579 | 0.0764 | 0.0211 |
| 16 | 0.322856 | 0.929333 | 0.0906 | 0.0258 |
| 17 | 0.309127 | 0.905583 | 0.1079 | 0.0341 |
| 18 | 0.297472 | 0.87862 | 0.1345 | 0.0469 |
| 19 | 0.269315 | 0.852335 | 0.1627 | 0.0638 |
| 20 | 0.254644 | 0.82635 | 0.2085 | 0.0957 |
| 21 | 0.245588 | 0.800175 | 0.2658 | 0.1421 |
| 22 | 0.239413 | 0.774751 | 0.3474 | 0.2236 |
| 23 | 0.226655 | 0.746126 | 0.5 | 0.3761 |
| 24 | 0.156272 | 0.715491 | 1 | 1 |
| 25 | 0.140951 | 0.679131 |  |  |

Note: The number of factors to be extracted based on each method is shown in bold.
