# Supplementary material for: The development of the Internal Resource Perception Scale: Validity and reliability
Source: PLoS One. 2026 Apr 29;21(4):e0348075. doi: 10.1371/journal.pone.0348075 (PMC13127970; doi:10.1371/journal.pone.0348075)
Supplement: S9 Table — (DOCX) [file pone.0348075.s009.docx]

**S9 Table. Factor loadings of the 25-item IRPS derived from the third round of EFA**

| **Resources**  **“I am…”** | Factor loadings | | | | |
| --- | --- | --- | --- | --- | --- |
|  | 3-factor model (alternative model) | | | 2-factor model (parallel analysis) | |
|  | 1 | 2 | 3 | 1 | 2 |
| loving | **.952** | .060 | -.182 | **.879** | -.107 |
| caring | **.889** | -.022 | .018 | **.941** | -.110 |
| easy-going | **.836** | .138 | -.207 | **.745** | -.030 |
| empathetic | **.834** | -.054 | .095 | **.933** | -.108 |
| conscientious | **.742** | -.084 | .221 | **.910** | -.077 |
| humble | **.640** | -.074 | .246 | **.814** | -.043 |
| faithful | **.543** | -.045 | .308 | **.743** | .024 |
| fair | **.532** | .015 | .270 | **.708** | .070 |
| free-spirited | **.529** | .147 | .120 | **.614** | .143 |
| analytical | **.325** | .134 | .316^a^ | **.506** | .237 |
| creative | .050 | **.801** | -.096 | -.042 | **.766** |
| positive | .132 | **.764** | -.106 | .041 | **.715** |
| determined | -.066 | **.728** | .182 | -.018 | **.835** |
| flexible | .060 | **.711** | -.104 | -.031 | **.671** |
| receptive | .214 | **.684** | -.183 | .091 | **.589** |
| rational | -.059 | **.670** | .176 | -.010 | **.773** |
| deliberate | -.221 | **.668** | .323 | -.101 | **.852** |
| courageous | -.058 | **.664** | .125 | -.035 | **.743** |
| enthusiastic | .027 | **.660** | .092 | .038 | **.715** |
| responsible | .020 | -.009 | **.775** | **.435** | .321^a^ |
| discipline | .041 | -.051 | **.771** | **.455** | .276^a^ |
| patient | .116 | .004 | **.688** | **.488** | .289^a^ |
| organized | .003 | .111 | **.632** | .338^a^ | **.382** |
| reliable | .184 | .079 | **.530** | **.474** | .289^a^ |
| intelligent | .123 | .211 | **.466** | .369^a^ | **.403** |
| % variance explained | 48.0% | 7.8% | 3.6% | 47.9% | 7.8% |

Note: Primary factor loadings are shown in bold.

^a^ Alternative absolute factor loading > 0.3 or a difference between the primary and alternative loadings > 0.2
