# Supplementary material for: The development of the Internal Resource Perception Scale: Validity and reliability
Source: PLoS One. 2026 Apr 29;21(4):e0348075. doi: 10.1371/journal.pone.0348075 (PMC13127970; doi:10.1371/journal.pone.0348075)
Supplement: S10 Table — (DOCX) [file pone.0348075.s010.docx]

**S10 Table. Model fitting indices of the 25-item IRPS**

| Model | χ² p-value | RMSEA [90% CI] | CFI | TLI | SRMR | BIC | % variance explained |
| --- | --- | --- | --- | --- | --- | --- | --- |
| **4-factor** | **< .001** | **.071 [.067, .076]** | **.924** | **.916** | **.044** | **21516** | **62.4** |
| 3-factor | < .001 | .084 [.080, .089] | .893 | .882 | .054 | 21787 | 59.5 |
| 2-factor | < .001 | .102 [.097, .106] | .843 | .828 | .078 | 22245 | 55.6 |
| 1-factor | < .001 | .133 [.129, .138] | .729 | .704 | .091 | 23296 | 47.6 |

Note: The best-fit model is shown in bold.
