# Supplementary material for: The development of the Internal Resource Perception Scale: Validity and reliability
Source: PLoS One. 2026 Apr 29;21(4):e0348075. doi: 10.1371/journal.pone.0348075 (PMC13127970; doi:10.1371/journal.pone.0348075)
Supplement: S11 Table — (DOCX) [file pone.0348075.s011.docx]

**S11 Table. Factor correlations and Fornell-Larcker Criterion**

| Factor | 1 | 2 | 3 | 4 | Discriminant validity met? |
| --- | --- | --- | --- | --- | --- |
| 1 | **0.87** |  |  |  | Yes |
| 2 | 0.64 | **0.80** |  |  | Yes |
| 3 | 0.80 | 0.72 | **0.85** |  | Yes |
| 4 | 0.76 | 0.70 | 0.73 | **0.88** | Yes |

Note: The square root of AVE values is shown in bold on the diagonal. The factor correlations are shown in the non-diagonal elements.
