# Supplementary material for: The development of the Internal Resource Perception Scale: Validity and reliability
Source: PLoS One. 2026 Apr 29;21(4):e0348075. doi: 10.1371/journal.pone.0348075 (PMC13127970; doi:10.1371/journal.pone.0348075)
Supplement: S12 Table — (DOCX) [file pone.0348075.s012.docx]

**S12 Table. Factor loadings of the 25-item IRPS based on the bifactor model**

| **Resources**  **“I am…”** | General factor | Specific factor 1 | Specific factor 2 | Specific factor 3 | Specific factor 4 |
| --- | --- | --- | --- | --- | --- |
| loving | .675 | .651 |  |  |  |
| caring | .751 | .588 |  |  |  |
| empathic | .785 | .500 |  |  |  |
| easy-going | .654 | .506 |  |  |  |
| conscientious | .812 | .438 |  |  |  |
| humble | .742 | .373 |  |  |  |
| faithful | .764 | .306 |  |  |  |
| fair | .771 | .292 |  |  |  |
| free-spirited | .750 | .241 |  |  |  |
| positivity | .625 |  | .527 |  |  |
| determined | .688 |  | .559 |  |  |
| creative | .603 |  | .521 |  |  |
| flexible | .564 |  | .506 |  |  |
| rational | .644 |  | .523 |  |  |
| enthusiastic | .629 |  | .492 |  |  |
| deliberate | .625 |  | .515 |  |  |
| receptive | .581 |  | .466 |  |  |
| courageous | .599 |  | .465 |  |  |
| responsible | .771 |  |  | .444 |  |
| discipline | .747 |  |  | .394 |  |
| patient | .791 |  |  | .292 |  |
| reliable | .792 |  |  | .289 |  |
| analytical | .775 |  |  |  | .379 |
| intelligent | .787 |  |  |  | .550 |
| organized | .736 |  |  |  | .342 |
| ω | .848 | .217 | .360 | .141 | .190 |
