# Supplementary material for: The development of the Internal Resource Perception Scale: Validity and reliability
Source: PLoS One. 2026 Apr 29;21(4):e0348075. doi: 10.1371/journal.pone.0348075 (PMC13127970; doi:10.1371/journal.pone.0348075)
Supplement: S13 Table — (DOCX) [file pone.0348075.s013.docx]

**S13 Table. Item reliability statistics within each factor of the 25-item IRPS**

|  | Item-total correlation | Item-rest correlation | Cronbach’s alpha if the item is deleted |
| --- | --- | --- | --- |
| **Factor 1** |  |  | **.94*** |
| loving | .85 | .80 | .93 |
| caring | .88 | .85 | .93 |
| empathetic | .88 | .84 | .93 |
| easy-going | .79 | .73 | .93 |
| conscientious | .87 | .83 | .93 |
| humble | .81 | .76 | .93 |
| faithful | .79 | .72 | .94 |
| fair | .77 | .72 | .93 |
| free-spirited | .76 | .68 | .94 |
| **Factor 2** |  |  | **.92*** |
| positive | .80 | .73 | .90 |
| creative | .79 | .72 | .90 |
| determined | .83 | .78 | .91 |
| flexible | .72 | .64 | .91 |
| enthusiastic | .78 | .71 | .91 |
| receptive | .72 | .64 | .91 |
| rational | .79 | .72 | .91 |
| deliberate | .80 | .73 | .91 |
| courageous | .76 | .68 | .91 |
| **Factor 3** |  |  | **.87*** |
| responsible | .86 | .75 | .81 |
| disciplined | .85 | .71 | .83 |
| patient | .86 | .72 | .83 |
| reliable | .81 | .68 | .84 |
| **Factor 4** |  |  | **.86*** |
| analytical | .88 | .72 | .77 |
| intelligent | .89 | .76 | .81 |
| organized | .88 | .71 | .82 |

* Cronbach’s alpha of each factor

.
