# Supplementary material for: The development of the Internal Resource Perception Scale: Validity and reliability
Source: PLoS One. 2026 Apr 29;21(4):e0348075. doi: 10.1371/journal.pone.0348075 (PMC13127970; doi:10.1371/journal.pone.0348075)
Supplement: S1 File — (DOCX) [file pone.0348075.s016.docx]

**Internal Resource Perception Scale**

Please answer to what extent you have these resources by placing an X in the box that best describes how you are.

| **Item** | **Resources**  (‘I am…’) | Not at all  (0) | A little  (1) | Quite a bit  (2) | Quite a lot  (3) | A lot  (4) |
| --- | --- | --- | --- | --- | --- | --- |
| 1 | loving |  |  |  |  |  |
| 2 | caring |  |  |  |  |  |
| 3 | empathetic |  |  |  |  |  |
| 4 | easy-going |  |  |  |  |  |
| 5 | conscientious |  |  |  |  |  |
| 6 | humble |  |  |  |  |  |
| 7 | faithful |  |  |  |  |  |
| 8 | fair |  |  |  |  |  |
| 9 | free-spirited |  |  |  |  |  |
| 10 | positive |  |  |  |  |  |
| 11 | creative |  |  |  |  |  |
| 12 | determined |  |  |  |  |  |
| 13 | flexible |  |  |  |  |  |
| 14 | enthusiastic |  |  |  |  |  |
| 15 | receptive |  |  |  |  |  |
| 16 | rational |  |  |  |  |  |
| 17 | deliberate |  |  |  |  |  |
| 18 | courageous |  |  |  |  |  |
| 19 | responsible |  |  |  |  |  |
| 20 | disciplined |  |  |  |  |  |
| 21 | patient |  |  |  |  |  |
| 22 | reliable |  |  |  |  |  |
| 23 | analytical |  |  |  |  |  |
| 24 | intelligent |  |  |  |  |  |
| 25 | organized |  |  |  |  |  |
